# Ubiquitylation at the Fork: Making and Breaking Chains to Complete DNA Replication

**DOI:** 10.3390/ijms19102909

**Published:** 2018-09-25

**Authors:** Maïlyn Yates, Alexandre Maréchal

**Affiliations:** Department of Biology, Université de Sherbrooke, Sherbrooke, QC J1K 2R1, Canada; mailyn.yates@usherbrooke.ca

**Keywords:** DNA replication stress, genome stability, ubiquitin, replication fork restart, translesion synthesis, template-switching, homologous recombination, Fanconi Anemia

## Abstract

The complete and accurate replication of the genome is a crucial aspect of cell proliferation that is often perturbed during oncogenesis. Replication stress arising from a variety of obstacles to replication fork progression and processivity is an important contributor to genome destabilization. Accordingly, cells mount a complex response to this stress that allows the stabilization and restart of stalled replication forks and enables the full duplication of the genetic material. This response articulates itself on three important platforms, Replication Protein A/RPA-coated single-stranded DNA, the DNA polymerase processivity clamp PCNA and the FANCD2/I Fanconi Anemia complex. On these platforms, the recruitment, activation and release of a variety of genome maintenance factors is regulated by post-translational modifications including mono- and poly-ubiquitylation. Here, we review recent insights into the control of replication fork stability and restart by the ubiquitin system during replication stress with a particular focus on human cells. We highlight the roles of E3 ubiquitin ligases, ubiquitin readers and deubiquitylases that provide the required flexibility at stalled forks to select the optimal restart pathways and rescue genome stability during stressful conditions.

## 1. Introduction

Precise and thorough replication of the genome is a pre-requisite for cell proliferation and the faithful transmission of genetic information to the progeny of all living organisms. In humans, this is a complex and difficult task considering the sheer number of bases that must be accurately replicated to produce an adult person composed of an estimated >10^13^ cells [1]. To complexify matters, a variety of obstacles can impede replication fork progression and processivity and create circumstances that lead to point mutations and rearrangements. These situations include repetitive and/or secondary-structure prone DNA sequences, DNA lesions, RNA:DNA hybrids, insufficient nucleotide levels, oncogene activation and many other replication hurdles that are collectively referred to as DNA replication stress [2,3].

**Figure 1:**
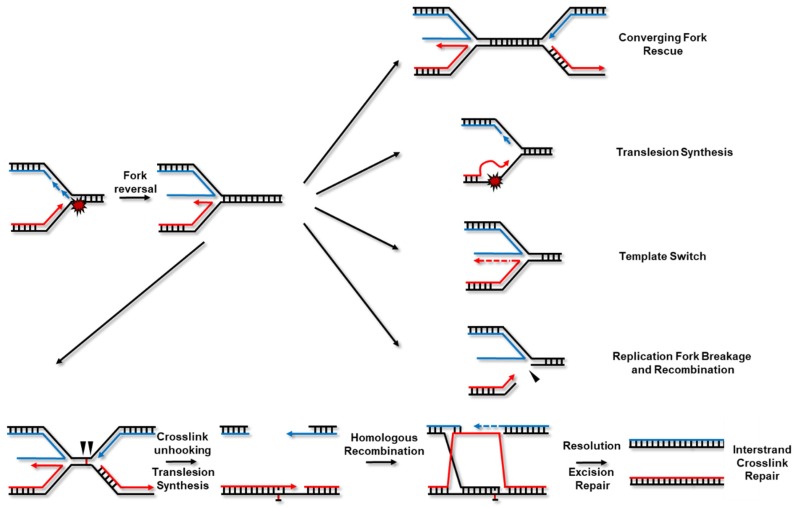
Replication fork Reversal and Restart Pathways. Stalled forks are rapidly reversed into four-branched structures by the combined activities of multiple DNA helicases. Stalled forks can be rescued by a converging fork arising from a nearby-fired origin or by activation of a local dormant origin by the replication stress response. In the event of replication fork stalling due to damaged bases, error-prone translesion polymerases can replicate past the problematic lesions. Alternatively, stalled polymerases can use an undamaged template to support genome replication, most often this template is the newly-synthesized strand on the sister chromatid. Stalled forks can also be nucleolytically processed (isosceles triangle) to yield single-ended DSBs that are repaired by recombination-based pathways. The best characterized inter-strand cross-link repair mechanism requires the convergence of two replication forks at the lesion but replication-independent repair can also occur. Single replication forks frequently traverse cross-links which allows post-replicative repair of the lesion. Nucleases (isosceles triangles) incise a single DNA strand in 5′ and 3′ of the cross-link thereby creating a double-strand break (DSB) concomitantly with cross-link unhooking. Translesion synthesis proceeds past the unhooked cross-link and homologous recombination with the sister chromatid repairs the DSB.

Recent reports have highlighted a unifying early response to replication stress in mammalian cells in which stalled forks rapidly regress to form 4-branched structures reminiscent of chicken feet by re-annealing of parental strands and concomitant annealing of the nascent DNA strands ([4,5] and Figure 1). This appears to be a universal response as fork reversal is well documented in prokaryotic systems and was also detected upon topoisomerase I inhibition in yeast, mouse and human cells as well as in *Xenopus laevis* egg extracts [5,6,7]. Moreover, this response is induced by treatments with mild concentrations of many replication disruptors indicating that it may be an invariable event during replication stress [4]. The reversal of ongoing forks into 4-way Holliday-like junctions had been proposed as a strategy to bypass DNA damage for a long time but the development of methods to enrich replication intermediates coupled with electron microscopy provided a robust assay to visualize these transient structures in eukaryotic systems [8,9]. Following fork reversal which is mediated by a variety of different DNA helicases, fork restart can be enabled by different pathways including rescue by convergent forks, recruitment of translesion polymerases and template-switching. In response to inter-strand cross-links (ICLs) which block replisome progression, the Fanconi Anemia (FA) repair pathway is engaged to remove covalent bonds between DNA strands and complete DNA replication [10,11,12,13]. Stalled forks may also be processed by structure-specific endonucleases into single-ended double-stranded DNA breaks (DSBs) which can then be repaired by recombination-dependent pathways such as break-induced replication [14,15].

The rapid reversal of replication forks and the subsequent restart mechanisms are regulated by an organized cellular response called the Replication Stress Response (RSR). A number of essential proteins control DNA replication and activate the RSR when necessary to safeguard genome replication. Chief among these factors, is the highly abundant heterotrimeric single-stranded (ss) DNA-binding complex Replication Protein A (RPA; composed of RPA70, 32, 14) [16,17]. The RPA complex binds ssDNA via its 4 central oligonucleotide/oligosaccharide binding (OB)-fold domains (3 on RPA70 and 1 on RPA32) which occupy −30 nts/trimer when fully extended. The high avidity of RPA for ssDNA supports unperturbed DNA replication by protecting these fragile regions against enzymatic processing and by disrupting secondary DNA structures that could slow down or block DNA polymerases. Additionally, RPA contains two protein-protein interaction modules: the RPA70 N-terminal OB-fold domain and the winged helix domain at the C-terminus of RPA32. These features allow the RPA-ssDNA platform to orchestrate the recruitment and activation of a large number of DNA damage signaling and repair factors to maintain genome stability [18,19,20,21].

The RSR is switched on by the detection of a deoxyribonucleic structure composed of persistent RPA-ssDNA and an adjacent single-/double-stranded (ds) DNA junction [22,23,24]. This structure results at least in part from the functional uncoupling of the replicative DNA helicase and polymerases during replication stress and activates the ATR-ATRIP master checkpoint kinase [25,26]. A similar ATR-activating structure can also arise from DSB resection, conferring an unusual flexibility to ATR in the detection of genome destabilizing lesions [27]. ATR is brought onto RPA-ssDNA by an interaction between its obligate partner ATRIP and RPA [28,29]. Once there, it is activated by direct contact with proteins that possess ATR-activating domains (AAD). In human cells, two such ATR activators have been described thus far: TOPBP1 (Topoisomerase II Binding Protein 1) and ETAA1 (Ewing’s Tumor Associated Antigen 1) [30,31,32,33]. It is thought that the physical contact between ATR-ATRIP and the AAD domain of its activators induces conformational changes that lead to ATR activation. Once activated via auto-phosphorylation, ATR modifies a variety of RSR effectors including RPA itself and the downstream kinase CHK1 to prevent the firing of new replication origins, activate fork repair factors and inhibit cell cycle progression to support the recovery and eventual completion of DNA replication prior to mitosis entry [34,35,36,37,38]. In addition to ATR activation, the ability of RPA to interact with numerous other DNA replication, recombination and repair factors implicates it in virtually all sub-pathways of replication fork processing and restart during replication stress.

Another key platform for the early RSR is the homotrimeric DNA polymerase sliding clamp PCNA (Proliferating Cell Nuclear Antigen) [39,40]. During normal replication, the ring-shaped homotrimeric PCNA is loaded onto primer template junctions by the Replication Factor C complex and encircles dsDNA while interacting with DNA polymerases to enhance their processivity [41,42,43]. PCNA is also important to mediate polymerase switches during replication and is a critical regulator of Okazaki fragment processing. Similarly to RPA, PCNA exerts its cellular functions by interacting with myriad genome maintenance factors that often bind PCNA through a short conserved PIP (PCNA interacting-peptide; consensus sequence Q-x-x-[I/L/M/V]-x-x-[F/Y]-[F/Y]) making this platform an essential component of DNA replication and the RSR [44]. Because of their symmetrical architecture, PCNA and its bacterial equivalent the beta sliding clamp can in principle interact productively with multiple different partners simultaneously giving rise to the tool belt hypothesis [45]. The alternative to this model is the sequential interaction of PCNA with individual enzymes and experimental support for both models exists in Okazaki fragment maturation by the FEN1, POL δ and Ligase I enzymes [46,47]. During DNA replication stress, PCNA interacts with various DNA helicases and polymerases and plays key roles in fork reversal, template-switching, homologous recombination (HR) and translesion DNA synthesis to maintain genome stability [8,48,49].

Finally, the FA DNA repair pathway processes highly toxic inter-strand cross-links (ICLs). FA is a rare human syndrome characterized by bone marrow failure, cellular hypersensitivity to cross-linking agents, cancer predisposition and skeletal defects among a host of other clinical manifestations. The mutations of 22 DNA repair genes result in at least a subset of FA symptoms and the proteins that they encode are considered *bona fide* FA factors [12]. The critical platform of this pathway is the FANCD2/I complex which initially recognizes the lesion and coordinates the recruitment of endonucleases to unhook the cross-link. Following the unhooking reaction, translesion polymerases, HR and nucleotide excision repair effectors collaborate to remove the chemical adduct and complete DNA replication (Figure 1). More recently, several FA proteins were also shown to function in various aspects of the RSR indicating that at least some components of this pathway may play important genome maintenance roles beyond the repair of ICLs [50,51,52,53,54].

Recruitment and exchange of interacting partners on RPA, PCNA and FANCD2/I, particularly in response to various types of DNA damage is controlled by post-translational modifications (PTMs) [16,40,55,56]. For instance, hyper-phosphorylation of the N-terminus of RPA32 by the ATR, ATM and DNA-PK DNA damage kinases regulates its association with the MRN complex, the PALB2 HR protein and the PRP19 E3 ubiquitin ligase among others [57,58,59]. Ubiquitylation has also emerged as a prevalent PTM that occurs on a large number of genome maintenance factors in response to replication stress and DSBs ([60,61,62] and reviewed in [63,64,65,66]). Modifications of PCNA, FANCD2/I and more recently of RPA by ubiquitin and ubiquitin-like modifiers have been shown to control their functions in genome maintenance. Here, we review recent findings on the ubiquitin ligases, ubiquitin-binding genome guardians and de-ubiquitylases that play key roles on the main RSR platforms to faithfully complete DNA replication under adverse conditions.

## 2. Ubiquitylation on the RPA-ssDNA Platform

The first indication that the RPA complex is ubiquitylated upon DNA damage came from proteomics analyses which showed that RPA70, RPA32 and RPA14 are all modified by ubiquitin in UV-treated cells [61]. Subsequently, RPA ubiquitylation was also shown to be enhanced by camptothecin (CPT; a topoisomerase I inhibitor that creates DSBs at ongoing forks), hydroxyurea (HU), the DNA polymerase inhibitor aphidicolin and the cross-linking agent mitomycin C but not by γ-irradiation (IR) indicating that this modification is particularly relevant to the RSR [21,67,68]. Two E3 ubiquitin ligases function on RPA-ssDNA and control RPA ubiquitylation during replication stress: PRP19 and RFWD3 [21,59,67,68,69].

### 2.1. PRP19, at the Intersection of mRNA Maturation and Genome Maintenance

PRP19 is a multifunctional and essential U-BOX family E3 ubiquitin ligase best known for its role as an evolutionarily conserved pre-mRNA processing factor that functions within the PRP19/CDC5L core complex, an important spliceosome co-factor composed of PRP19, CDC5L, PLRG1 and BCAS2 [70,71,72,73,74]. In addition to its U-BOX E3 ligase domain, PRP19 also contains a central coiled-coil domain which allows its tetramerization and a C-terminal substrate recognition WD40-repeat module [69,75,76]. It was first discovered in yeast as a mutant that accumulates intron-containing pre-mRNA at non-permissive temperature and was later shown to physically associate with the spliceosome and promote its activation [77,78,79]. Mechanistically, PRP19 is part of a ubiquitylation/de-ubiquitylation cycle that targets the PRP3 subunit of the U4/U6 snRNP with non-degradative K63-linked ubiquitin chains to promote spliceosome maturation and productive rounds of mRNA splicing [80].

*Saccharomyces cerevisiae*, *PRP19* is also known as *PSO4* and was independently found in a genetic screen for photoactivated psoralen-sensitive mutants suggesting that it may play a role in ICL repair [81]. Depletion of the PRP19/CDC5L complex also inhibits psoralen cross-link repair *in vivo* in human cells [82]. Additional early clues for a DNA repair function of PRP19 came from the observation that its overexpression enhances the resistance to genotoxic insults and increases the replication lifespan of umbilical vein endothelial cells [83]. Knockdown (KD) of the core subunits of the PRP19/CDC5L complex also results in sensitivity to the replication stress-inducing agents mitomycin C, UV and HU [84,85]. Support for a more direct link between the PRP19 complex and the RSR came from the discovery that the complete PRP19/CDC5L core complex relocates onto RPA-ssDNA upon replication stress or at resected DSBs [21,69,86]. On RPA-ssDNA, the PRP19 complex promotes ATR activation. Indeed KD of PRP19, CDC5L, PLRG1 or BCAS2 all strongly decrease RSR signaling by ATR and the recruitment of this master checkpoint kinase to stalled forks as measured by phosphorylation of its substrates (RPA and/or CHK1) and by ATRIP foci formation [21,69,84,86]. Depletion of PRP19 and other splicing factors was also shown to impede DSB resection which may contribute to ATR-activation and RPA phosphorylation defects [21,69,87,88]. Furthermore, PRP19 complex KD impedes replication fork restart and HR indicative of extensive roles in the RSR [21,87,89]. Importantly, a WD40-repeat point mutant defective for the PRP19-RPA interaction but still able to form the PRP19-CDC5L splicing complex is unable to support ATR activation, HR and timely repair of collapsed replication forks, demonstrating the dual roles of PRP19 in mRNA processing and the RSR [21,59]. A deletion of the U-BOX domain also impedes ATR activation and HR linking them to PRP19-mediated ubiquitylation. Mechanistically, PRP19 KD decreases RPA70 and RPA32 ubiquitylation upon CPT treatment and this can be complemented by the re-expression of WT PRP19 but not by ubiquitin ligase or RPA-binding mutants [21,59]. More recent data indicates that RPA ubiquitylation also depends on PLRG1 which is required to activate the E3 ligase activity of the PRP19/CDC5L core complex [69]. Thus, in response to replication stress, the PRP19 complex transforms into a sensor of RPA-ssDNA and functions as a ubiquitin ligase on RPA-ssDNA to promote RSR signaling and replication fork repair.

### 2.2. RFWD3, a Novel Fanconi Anemia Player on RPA-ssDNA

RFWD3 is a RING (Really Interesting New Gene) domain E3 ubiquitin ligase that contains a coiled-coil domain and a WD40-repeat substrate-binding module. The first link between RFWD3 and the DNA damage response came from the demonstration that RFWD3 works together with the MDM2 E3 ligase to control the length of ubiquitin chains polymerized onto p53. In this context, RFWD3-mediated ubiquitylation is non-degradative and promotes the stability of p53 in response to DSBs [90]. Initial evidence for an implication of RFWD3 in the RSR came when it was isolated as an RPA32 interactor and shown to be required for optimal RPA and CHK1 phosphorylation [91,92]. The magnitude of the CHK1 phosphorylation defect induced by RFWD3 KD appears to be somewhat cell-type dependent perhaps due to variations in ATR activation thresholds [67,91,92]. However, in line with an ATR-activating role for RFWD3, its downregulation leads to increased new origin firing during replication stress, as might be expected if the S-phase checkpoint is defective [67]. This particular function of RFWD3 in ATR signaling appears to be independent from its ubiquitin ligase activity but to require its interaction with RPA [68]. Early phenotypic characterization also noted the inability of RFWD3-depleted cells to resolve HU-induced RPA and RAD51 foci in a timely manner suggesting that some step(s) in replication fork restart might depend on this E3 ubiquitin ligase [92]. Accordingly, RFWD3 depletion or mutation renders cells sensitive to a variety of replication stressors including CPT, mitomycin C, cisplatin and olaparib with milder sensitivities to HU and γ-irradiation [68,91,92,93,94].

A breakthrough came from the discovery that RFWD3 also acts as a ubiquitin ligase on RPA70 and RPA32 during replication stress and promotes HR repair of DSBs and stalled replication forks [67]. Downregulation of RFWD3 abrogrates ubiquitylation of RPA in response to HU, 4-NQO (4-nitroquinoline), mitomycin C and CPT treatments [59,67,68]. Some of the phenotypes seen upon RFWD3 depletion could be recapitulated by a RPA32 ubiquitylation mutant (K37/38R) which exhibited replication fork repair defects. Nevertheless, this mutant was still able to support HR at stalled forks, possibly due to the fact that at least 18 potentially interchangeable lysines are modified on the RPA complex, which makes it difficult to create fully ubiquitylation-defective RPA [67]. Interestingly, a RPA32 C-terminal winged-helix domain mutant that cannot interact with RFWD3 mutant also had impaired phosphorylation suggesting crosstalk between these 2 PTMs on RPA-ssDNA. In response to replication stress, it does not appear that ubiquitylation mediated by UV or 4-NQO treatment is degradative as no increase in ubiquitylated RPA species or in total RPA levels could be observed in response to proteasome inhibition [67].

Another important development was recently made with the implication of RFWD3 as a novel FA pathway factor and the discovery that RFWD3-mediated ubiquitylation is an important contributor to ICL repair [68,93,94]. Mechanistically, it was found that both RPA and the RAD51 HR recombinase directly interact with RFWD3 and are ubiquitylated in response to mitomycin C [68]. In contrast to the ubiquitylation induced by short term UV or 4-NQO treatments, long-term mitomycin C treatment destabilizes RPA32 and RAD51 proteins in a RFWD3-dependent manner [67,68]. Enhanced levels of RPA32 and RAD51 ubiquitylation induced by mitomycin C were observed following proteasome or VCP (Valosin-containing protein/p97) inhibition and the turnover of RPA32 in mitomycin C-induced foci was decreased upon RFWD3 KD but not in response to PRP19 depletion, indicating a specific role of RFWD3 in this genotoxic context. *In vitro*, ubiquitylation of RPA70 and 32 and of RAD51 decreased their affinity for ssDNA. RPA32 or RAD51 ubiquitylation mutants also showed some impairment in turnover rates as well as defective HR repair and sensitivity to ICL agents implicating their modification directly in these processes. The loading of the RAD54 SWI2/SNF2 ATPase motor protein and the MCM8 helicase were perturbed by RFWD3 KO indicating extensive defects in the later steps of ICL repair.

RFWD3 was also directly implicated in FA when a patient was found with a frameshift leading to premature termination on one RFWD3 allele and a missense mutation (I639K) in the WD40-repeat domain of the other. This patient presented FA symptoms including absent thumbs, pan-cytopenia of the bone marrow and hypersensitivity of cells to mitomycin C and diepoxybutane. Chromosomal breakage, G2-arrest and cell death induced by mitomycin C treatment of patient fibroblasts could be complemented by re-expression of WT RFWD3 establishing this ubiquitin ligase as a novel FA factor [68,93,94]. Mutation of I639K and other residues on the WD40-repeat domain abrogates the recruitment of RFWD3 to sites of damage, its interaction with RPA and RAD51, the ubiquitylation of these substrates, and HR repair. Altogether these studies convincingly implicate RFWD3 in the regulation of HR that occurs towards the end of ICL repair [68,93,94].

### 2.3. Potential Interplay between PRP19 and RFWD3 on RPA-ssDNA 

**Figure 2:**
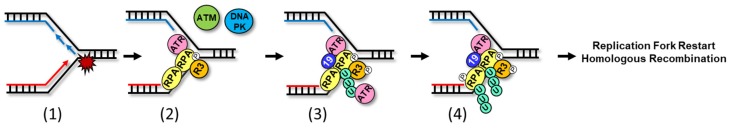
Ubiquitylation on the RPA-ssDNA platform during Replication Stress. (**1**) Fork uncoupling leads to RPA-ssDNA production which is constitutively associated with the RFWD3 (R3) ubiquitin ligase; (**2**) RPA hyper-phosphorylation by ATR, ATM and DNA-PK enhances the recruitment of PRP19 (19); (**3**) PRP19 and RFWD3 poly-ubiquitylate the RPA complex. K63-linked chains help tether ATR-ATRIP to RPA-ssDNA; (**4**) this produces a feed-forward loop that results in the spreading of RPA phosphorylation and ubiquitylation across RPA-ssDNA filaments. These modifications stimulate RSR signaling, fork restart and homologous recombination.

How could RFWD3 and PRP19 collaborate to regulate the RSR? Whereas RFWD3 is constitutively associated with the RPA32 C-terminal winged-helix domain, the PRP19 complex is specifically recruited to RPA-ssDNA during replication stress [21,59,62]. Upon damage, PRP19 also interacts with RFWD3, perhaps by joining it on the RPA complex as PRP19 was shown to associate with RPA70 whereas RFWD3 is tethered to the C-terminus of RPA32 [21,59,86,93]. The PRP19-RPA interaction is strongly stimulated by CPT treatment which creates DSB at replication forks. This CPT-triggered PRP19-RPA interaction is decreased by ATR kinase inhibition and completely abrogated when ATR, ATM and DNA-PK are jointly inactivated [21,59]. All 3 of these DNA damage response kinases phosphorylate their own specific substrates in response to DNA damage but they act together on the RPA32 N-terminus to promote its full hyper-phosphorylation during replication-associated DSBs [55,95]. Interestingly, an RPA32 mutant that cannot be phosphorylated still binds RFWD3 but cannot interact with PRP19 or be ubiquitylated in response to CPT. Reciprocally, a PRP19 point mutant that cannot interact with phosphorylated RPA is unable to support CPT-mediated RPA ubiquitylation [59,69]. Part of the ubiquitylation occurring on RPA occurs as non-degradative K63-linked chains, which are important regulators of protein-protein interactions [21,67,96,97]. *In vitro*, ATRIP exhibits affinity for K63-linked ubiquitin chains that may favor ATR-ATRIP recruitment onto ubiquitylated RPA-ssDNA. This led to the proposal of a feed-forward loop for ATR activation in which RPA hyper-phosphorylation by ATR, ATM and DNA-PK enhances its interaction with PRP19 and its ubiquitylation by both RFWD3 and PRP19 which may further promote fork restart and HR (Figure 2, [21,59]). In this context, the E3 ubiquitin ligase activity of PRP19 on RPA-ssDNA would promote ATR signaling, HR and fork restart whereas RFWD3 would be geared more towards fork restart and HR. Use of different sites or types of ubiquitin chains by each ligase on RPA may explain their differential requirements in these aspects of the RSR. Interestingly, a notable exception to this model is for mitomycin C treatment which increases the RFWD3-RPA interaction [93]. This enhanced recruitment of RFWD3 to RPA in response to ICLs may explain the degradative nature of RFWD3-mediated ubiquitylation in this context. Additional work will be required to understand the mechanisms which control the balance of degradative and non-degradative ubiquitylation on RPA-ssDNA.

Another potential point of intersection between PRP19 and RFWD3 could be at the level of RFWD3 phosphorylation. It was shown that RFWD3 is phosphorylated by ATM/ATR in response to replication stress and this phosphorylation is required for mitomycin C resistance as well as for RPA ubiquitylation [68,90,93]. Because of PRP19’s role in ATR activation it is possible that some of the negative impact of PRP19 depletion on RPA ubiquitylation may be through a decrease in the stimulatory phosphorylation of RFWD3 during the RSR. It is also possible that these two ligases may confer some flexibility in the response to specific genotoxic circumstances that may lead to RPA ubiquitylation. In this regard, it has been shown that RPA is also SUMOylated (small ubiquitin-like modifier) in response to DSBs. SUMO-RPA enhances RAD51 recruitment and promotes HR repair of the breaks [98]. It was suggested that SUMO-RPA may be recognized and targeted by the RNF4 SUMO-targeted ubiquitin ligase (STUbL) to facilitate RPA removal from ssDNA and promote HR-mediated repair of breaks [98,99]. However, in contrast to the PRP19 and RFWD3 ligases, direct ubiquitylation of RPA by RNF4 remains to be demonstrated. Under different genotoxic circumstances, RFWD3, PRP19 and RNF4 may be called into action to modify RPA and regulate its many genome maintenance functions. Finally, as RPA-ssDNA orchestrates the recruitment of multiple genome guardians during the RSR, the ubiquitylation of additional substrates at stalled forks may explain some of the phenotypic differences observed in PRP19- and RFWD3-deficient cells as well. How exactly, these different E3 ligases work together on RPA-ssDNA will be an interesting avenue of future research.

## 3. Ubiquitylation on the PCNA Platform

Besides the RPA-ssDNA platform, PCNA is essential for DNA replication, DNA damage tolerance and genome stability. PCNA is a homotrimeric DNA polymerase processivity factor. With its ring-shaped structure, PCNA encircles DNA to form a sliding clamp and acts as a protein loading scaffold during unperturbed DNA replication. In addition to its critical roles during replication, PCNA serves as a recruitment platform for genome maintenance proteins and choreographs their activities within multiple repair pathways (reviewed in [39,40]). Similarly to the RPA-ssDNA platform, studies revealed that ubiquitylation and/or SUMOylation of PCNA are important for cell survival in response to DNA damaging agents that block replication fork progression such as UV, methyl methanesulfonate, mitomycin C or HU [100,101]. Whereas DNA damaging agents that cause fork stalling lead to PCNA ubiquitylation, others such as CPT or bleomycin that cause DSBs do not, underlining the relevance of PCNA ubiquitylation for the restoration of blocked replication forks. 

In response to replication fork stalling or during normal S-phase, mono- or poly-ubiquitylation and/or SUMOylation on PCNA occurs on a major site, the highly conserved lysine 164 (K164) residue [102,103,104,105]. PCNA mono-ubiquitylation in yeast and in mammals is mainly catalyzed by the conserved Rad18(E3)/Rad6(E2) complex [106]. Poly-ubiquitylation of PCNA by addition of K63-linked ubiquitin chains onto mono-ubiquitylated PCNA depends on the heterodimeric complex Ubc13-Mms2 (E2) first discovered in yeast (UBC13-UEV1 in humans) in association with the Rad5 ubiquitin ligase (HLTF or SHPRH in humans) [103,107,108]. In yeast, PCNA is also SUMOylated to a lesser extent on K127 and additional sites are also found in human cells [102,109]. In yeast, PCNA SUMOylation involves Ubc9 (E2) and Siz1 and Siz2 (E3a) whereas the SUMO E3 in humans in currently unknown [102,103,104,110]. In human cells, endogenous DNA damage also promotes K164 PCNA mono-ubiquitylation by CRL4^Cdt2^, instead of RAD18 [111]. PCNA ubiquitylation can also be stimulated by accessory co-factors such as SIVA1, a PCNA and RAD18-interacting protein [112]. A notable exception to K164-targeted ubiquitylation was discovered in DNA ligase I-defective yeast cells. In this context, deficient Okazaki fragment ligation leads to ubiquitylation of PCNA at K107 at non-permissive temperatures to promote DNA damage signaling [113]. Although the specific lysine residue remains to be determined, it seems that this alternative modification is also conserved in humans [113,114].

A functional link between the RPA-ssDNA and PCNA ubiquitylation has been established in yeast where RPA-ssDNA serves as a recruitment platform for Rad18-Rad6 at stalled forks and controls PCNA ubiquitylation [115]. In humans, some evidence also suggests that RPA-ssDNA regulates PCNA ubiquitylation further supporting close collaboration between both platforms in genome maintenance [101].

**Figure 3:**
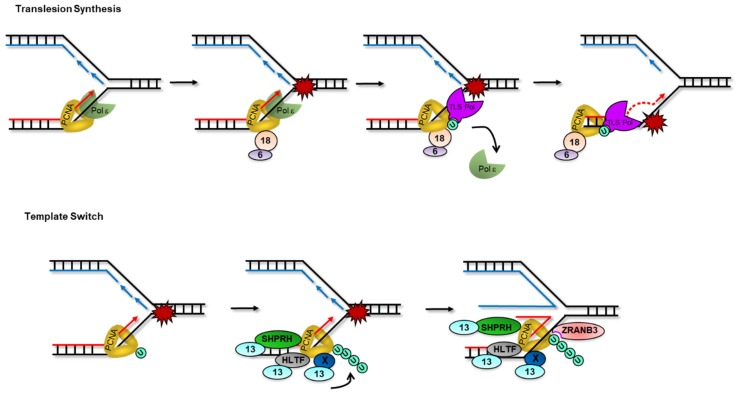
Ubiquitylation on PCNA during Replication Stress. When forks encounter DNA lesions, PCNA is either mono-ubiquitylated to engage translesion synthesis or poly-ubiquitylated to trigger lesion bypass by template switching. RAD18(18)-RAD6(6) is recruited and mono-ubiquitylates PCNA at K164. Mono-ubiquitylated PCNA is recognized by specialized TLS polymerases that contain PCNA-interacting and ubiquitin-binding domains (e.g., POL η, κ, ι, REV1). TLS polymerases replace replicative polymerases to continue DNA synthesis across the lesions. Addition of K63-linked ubiquitin chains on mono-ubiquitylated PCNA is mediated by the UBC13-UEV1 (13) E2 complex which functions with either HLTF, SHPRH and/or currently unknown E3 ligases (X). Poly-ubiquitylated PCNA recruits fork remodeler ZRANB3 via its ubiquitin binding zinc finger to allow fork regression.

The modification of PCNA by ubiquitin and ubiquitin-like molecules controls repair pathway choice at replication-blocking obstacles. Two major pathways mediate DNA damage tolerance mechanisms to allow replication completion in the presence of damage: Translesion synthesis (TLS) and template switching (TS) (Figure 3 and reviewed in [116]). While PCNA mono-ubiquitylation promotes TLS that constitutes to some extent an error-prone lesion bypass mode, PCNA poly-ubiquitylation promotes TS, an error-free recombination-based repair pathway.

### 3.1. PCNA Mono-Ubiquitylation and Translesion Synthesis 

TLS is a highly conserved repair mechanism that uses low fidelity polymerases to replicate damaged DNA segments thus allowing completion of DNA replication (reviewed in [116]). During TLS, DNA polymerase switches happen during which replicative polymerases (i.e., Pol δ and Pol ε B-family polymerases) are replaced by specialized TLS polymerases that are capable of inserting nucleotides opposite lesions [117,118,119]. Once nucleotide incorporation opposite the lesion is made, the TLS patch is then extended by the same or another TLS polymerase. The extension step can range from 5–60 nucleotides depending on the lesion and polymerase involved. The low processivity of TLS polymerases enables a final switch that brings back a replicative DNA polymerase to resume bulk genome replication.

The best characterized TLS enzymes include members of the Y-family of DNA polymerases Pol η (eta; POLH/XPV/RAD30A), Pol ι (iota; POLI/RAD30B), Pol ҡ (kappa; POLK/DINB1) and REV1 along with the B-family polymerase Pol ζ (zeta), a tetrameric complex composed of the catalytic subunit REV3L, REV7/MAD2L2/FANCV, POLD2 and POLD3 ([120,121,122,123] and reviewed in [124,125,126,127]). When compared with replicative polymerases, these specialized polymerases generally exhibit lower nucleotide incorporation fidelity due to a more spacious/flexible active site and lack exonucleolytic proofreading function. TLS polymerases each have distinct properties that make them either mutagenic or accurate depending on the type of lesion encountered. Care must thus be taken at the selection step during polymerase switch to pick the best enzyme for the job at hand. For example, POL η/XPV is the major TLS polymerase selected to bypass UV-induced *cis-syn* cyclobutane thymine-thymine dimers (CPD) in a largely error-free manner [128,129,130,131]. The importance of POL η/XPV in UV-induced damage repair is emphasized by the fact that its mutation in humans causes xeroderma pigmentosum, a syndrome characterized by extreme sensitivity to sunlight and a high incidence of skin cancer [132,133]. In the absence of POL η, TLS at CPDs is believed to be catalyzed by a different specialized polymerase which frequently incorporates incorrect nucleotides thereby generating mutations that promote cancer [134].

PCNA is the main conductor of polymerase switches during TLS and this is highly regulated by its mono-ubiquitylation. TLS polymerases of the Y-family POL η, POL ι and POL ҡ possess PIP motifs while REV1 interacts with PCNA via its BRCT (BRCA1 C Terminus) domain [135,136,137,138,139]. Besides these PCNA-interacting regions, the Y-family of TLS polymerases also possess evolutionarily conserved ubiquitin-binding motifs (UBM) and/or ubiquitin-binding zinc finger (UBZ) domains that mediate the recognition of mono-ubiquitylated PCNA [140,141]. These ubiquitin-binding domains are important for genome stability as exemplified by the requirement of the Pol η UBZ to restore normal response to UV in XP-V fibroblasts [142]. Despite strong experimental support that PCNA mono-ubiquitylation is required for maximal TLS in mammalian cells, this modification is not absolutely essential for this process to occur. KD of REV3L, POL η or REV1 in PCNA K164R mouse embryonic fibroblasts (MEFs) leads to increased UV sensitivity indicative of additional modes of recruitment for TLS polymerases at lesions [143]. These alternative recruitment modes vary; certain TLS polymerases such as POL η and POL ҡ interact with RAD18 to facilitate their access to DNA templates. Furthermore, REV1 binds ssDNA and primer termini and recruits different TLS polymerase partners including POL ζ, POL η, POL ι and POL κ to sites of damage. In essence, mono-ubiquitylated PCNA works with REV1 and its partner TLS polymerases to coordinate both steps of the TLS reaction: nucleotide insertion opposite the lesion and extension of the nascent strand [117,144,145,146].

### 3.2. PCNA Poly-Ubiquitylation and Template Switching 

TS is a damage avoidance repair mechanism based on recombination that uses the undamaged sister chromatid as the template to carry out limited DNA replication that allows the replication fork to bypass problematic lesions in a mostly error-free manner (reviewed in [147,148,149]). Even though the mechanistic details have not been completely worked out, TS at the fork is thought to happen after fork reversal. DNA synthesis would then allow the stalled nascent DNA to extend past the lesion on the undamaged switched template (Figure 1 and [147]). TS can also occur post-replicatively and initiate behind the fork at gapped lesions that were left behind [150,151].

In yeast, one of the major actors of this pathway is Rad5, a member of the SWI/SNF2 ATPase family which is also a RING domain E3 ubiquitin ligase. Rad5 mediates K63-linked PCNA poly-ubiquitylation with its partner E2 Ubc13-Mms2 to extend the mono-ubiquitylation product created by Rad18/Rad6 [102,103,108]. Moreover, the ATP-dependent helicase activity of Rad5 allows it to unwind and anneal nascent and parental strands together to promote replication fork reversal [107,152]. Both the ubiquitin ligase and ATPase activities of Rad5 were shown to be required for genome stability in response to a variety of different replication stressors [153,154,155].

Despite years of efforts, it is still unclear how PCNA poly-ubiquitylation in yeast promotes TS and error-free damage bypass. What is clear is that sister chromatid junction formation during TS requires PCNA SUMOylation and the Rad51 recombinase [156,157,158]. During an unperturbed S-phase, unscheduled recombination is actively counteracted when DNA replication is proceeding smoothly. Indeed, PCNA SUMOylation directly mediates the recruitment of the anti-recombinase/UvrD family helicase Srs2 which is equipped with SUMO-interacting (SIMs) and PIP-like motifs. On SUMO-PCNA, Srs2 minimizes Rad51-dependent recombination at ongoing forks by disturbing the formation of Rad51-ssDNA filaments [159,160,161,162,163,164]. The alternative clamp loader Elg1 is also tethered to SUMO-PCNA via SIMs and PIP-like motifs to promote PCNA unloading after Okazaki fragment maturation and this role is shared by the Elg1 homolog in human cells, ATAD5 [165,166,167,168]. Moreover, SUMO-PCNA positively regulates its damage-induced mono- and poly-ubiquitylation by recruiting the Rad6-Rad18 ligase which exhibits at least partial STUbL behavior on this platform. In this case however, the STUbL activity does not appear to be shared by the human RAD18 homolog [169]. 

How Rad51 comes into action at stalled replication forks in yeast to induce TS when necessary has recently been brought to light. It was found that the adaptor protein Esc2 possesses SUMO-like domains (SLDs) which interact with SIMs on Srs2. Esc2 can also bind directly to branched DNA structures *in vitro* and to stalled replication forks *in vivo*. At stalled forks, Esc2 and the STUbL Slx5-Slx8 increase the turnover of SUMOylated Srs2 thereby allowing Rad51 recruitment and local induction of recombination [170,171]. Downstream of PCNA poly-ubiquitylation and Rad51-mediated TS, the Sgs1 helicase, together with Top3 and Rmi1 (BLM/TOP3A/RMI1/2 in humans) dissolves recombination intermediates to promote the formation of non-crossover products [156,172]. Backup sister chromatid junction resolution is also provided by the Mus81-Mms4 complex with assistance by Esc2 [173,174,175].

In this context, the role of poly-ubiquitylated PCNA in promoting this cascade of events still remains a bit obscure. Thus far in yeast, the only known interactor of poly-ubiquitylated PCNA is the AAA^+^ ATPase Mgs1 [176]. Interestingly, *MGS1* exhibits synthetic lethality with *RAD6* and synthetic sickness with *RAD18* and *RAD5*, implicating it in the protection and repair of stalled replication forks [177]. Mgs1 interacts with poly-ubiquitylated PCNA via its UBZ domain. This domain allows Mgs1 to outcompete the Pol δ subunit Pol32 on PCNA which may be important for a variety of outcomes at stalled forks. However, a Mgs1 UBZ domain mutant still rescues synthetic lethality of *mgs1 rad18* mutants as well as the WT and the lack of DNA damage sensitivity of *MGS1* single mutants does not support a critical role for this protein in damage bypass [177,178]. The identification of poly-ubiquitylated PCNA-binding effector(s) that may promote TS in yeast will be a very significant step forward in our understanding of this error-free repair mechanism. Another scenario stems from the very recent finding that K63-linked poly-ubiquitin chains can bind to DNA [179]. This opens up the possibility that PCNA poly-ubiquitylation by itself may locally influence the architecture of stalled replication forks and perhaps help their eventual restart.

In human cells, a similar pathway appears to promote damage avoidance and two Rad5 functional homologues were identified: Helicase-like transcription factor (HLTF) and SNF2 histone-linker PHD-finger RING-finger helicase (SHPRH) [180,181]. Both SHPRH and HLTF present extensive structural and functional similarities with yeast Rad5 including RING domains that function with the E2 complex UBC13-MMS2 to poly-ubiquitylate PCNA in response to replication stress [180,181,182,183,184]. Additional E3 ligase(s) may also take part in PCNA poly-ubiquitylation in mammals as double HLTF/SHPRH mutant MEFs showed a decrease but not an abrogation of PCNA poly-ubiquitylation in response to UV [185]. *In vitro*, HLTF can carry out fork reversal and also complements the UV sensitivity of the *rad5* yeast mutant further supporting the notion that it acts as a Rad5 homolog to promote genome maintenance [181,186,187]. In addition to its SWI/SNF helicase domain, HLTF has a HIRAN (HIP116 and RAD5 N-terminus) region which functions as a substrate recognition module. The HIRAN domain is structurally similar to an OB-fold motif and mediates binding of HLTF at 3′ ssDNA ends to drive replication fork reversal [188,189,190,191]. This domain is not found in SHPRH which also cannot complement *rad5* mutant UV sensitivity suggesting that despite their common function in PCNA poly-ubiquitylation, HLTF and SHPRH may also have distinct roles in genome maintenance [184].

Little is known about how HLTF and SHPRH collaborate or how they split the work to regulate replicative lesion bypass. One possibility is that each ligase may be activated in response to different lesions or genotoxic circumstances. Supporting this idea, is the fact that HLTF and SHPRH were shown to play distinct roles in the response to UV or MMS respectively [192]. MMS induces HLTF degradation perhaps via auto-ubiquitylation and the formation of a RAD18-SHPRH-Pol κ complex that allows bypass of the lesions. Contrastingly, following UV irradiation HLTF but not SHPRH promotes the recruitment of Pol η which can bypass UV lesions in an error-free manner [192]. These roles of HLTF and SHPRH in controlling some aspects of TLS are also supported experimentally for the Rad6-Rad18-Rad5-Ubc13-Mms2 axis in fission yeast [193]. Interestingly, Pol η has a single ubiquitin-binding motif whereas Pol κ and other TLS polymerases have 2 [142]. It is possible that PCNA mono- and poly-ubiquitylation may be induced differentially depending on the source of replication stress. In turn, this may influence TLS polymerase selection to maximize error-free repair of lesions.

In human cells, the SWI/SNF2 family ATPase ZRANB3/AH2 (zinc finger, RAN-binding domain containing 3) is recruited onto poly-ubiquitylated PCNA and mediates replication fork reversal and restart. In addition to fork regression activity, ZRANB3 can dismantle D-loops *in vitro* and limit sister-chromatid exchange *in vivo* [194,195,196,197]. The recruitment of ZRANB3 to stalled forks requires a PIP and an APIM (AlkB2 PCNA interaction motif) motif that bind directly to PCNA, a NZF (NPL4 zinc finger) which recognizes K63-linked ubiquitin chains and a HNH nuclease domain [195,197,198,199]. RAD18 or UBC13 depletion impaired the retention but not the initial recruitment of ZRANB3 at UV stripes indicating that ZRANB3 may also be recruited in a PCNA ubiquitylation-independent manner to sites of damage. HLTF and ZRANB3 along with a number of other factors function as fork remodelers at stalled forks. One explanation for the requirement of many enzymes for this process may lie in the variety of structures created in response to different types of lesions [200]. It is also possible that different remodelers act in a sequential manner at different steps of the reversal reaction. In agreement with this hypothesis, SMARCAL1, HLTF or ZRANB3 KO all led to a complete abrogation of stalled fork degradation by the MRE11 nuclease in BRCA1/BRCA2 mutant cells suggesting that these enzymes are all necessary for fork reversal [196,201]. Importantly, PCNA ubiquitylation, UBC13 and the interaction between ZRANB3 and poly-ubiquitylated PCNA were recently shown to be required for fork reversal *in vivo*, suggesting a sequential model where the PCNA-RAD18-UBC13 system would promote the arrival of ZRANB3 to stalled forks and drive their remodeling to support genome stability [194]. Whether HLTF, SHPRH or perhaps another E3 ubiquitin ligase controls this process remains to be determined.

The WRN-interacting protein 1 (WRNIP1) AAA+ ATPase is the human homolog of Mgs1 and like its yeast counterpart, it can interact with ubiquitylated PCNA [202]. The localization of WRNIP1 to replication factories or micro-irradiation stripes depends on its UBZ domain which, *in vitro* shows preference for ubiquitin chains over single ubiquitin polypeptides [202,203,204]. WRNIP1 foci formation was decreased albeit not totally abrogated upon mutation of the K164 ubiquitin-acceptor site on PCNA suggesting that additional ubiquitylated proteins might tether WRNIP1 to sites of damage [205]. WRNIP1 is also able to bind directly to forked DNA structures resembling stalled forks and to interact with RAD18 [205,206]. WRNIP1 plays multiple roles at stalled forks which include ATM activation, suppression of sister chromatid exchange and the stabilization of RAD51 on ssDNA to prevent MRE11-mediated fork degradation during replication stress but whether these functions depend on its UBZ domain remains undisclosed at the moment [205,207,208].

Finally, in human cells, PCNA is also SUMOylated although at lower levels than in yeast [109,209]. Similar to the situation in yeast, SUMOylated human PCNA is bound by an anti-recombinase to prevent unscheduled recombination. Like its yeast equivalent Srs2, PARI (PCNA-associated recombination inhibitor) also contains a UvrD helicase domain, a SIM and a PIP box. PARI binds SUMOylated PCNA and limits recombination during ongoing DNA replication. *In vitro*, purified PARI can disrupt RAD51 nucleofilaments and its PIP and SIM domains were also shown to mediate CPT resistance [209]. PARI also has a helicase-independent but PCNA-interaction dependent role in the suppression of HR. In this latter study, RAD51 nucleofilament disruption by PARI was not detected but a defect in D-loop extension attributed to a competition between PARI and Pol δ for PCNA was reported [210]. Additionally, cells expressing a non-SUMOylatable K164R PCNA mutant still supported PARI foci indicating that other SUMO-modified proteins help tether PARI to stalled replication forks. Thus, SUMOylation of PCNA and other proteins during replication stress is an evolutionarily conserved strategy for the recruitment of effectors that prevent undesired recombination.

## 4. Ubiquitylation and the Fanconi Anemia Pathway

ICLs differ from most other replication stressors in that they completely block the progression of DNA replication on both strands of the parental duplex. Covalent bonds between both filaments of the double helix also obstruct any DNA transaction that requires strand separation. The FA repair pathway resolves these damaging lesions and ubiquitylation plays prominent roles in this process. Here we will provide a brief overview of this pathway and discuss recent discoveries on the importance of ubiquitin in the regulation of ICL repair. For additional details, there are numerous excellent detailed reviews on the FA machinery to which we refer readers [10,11,12,13].

**Figure 4:**
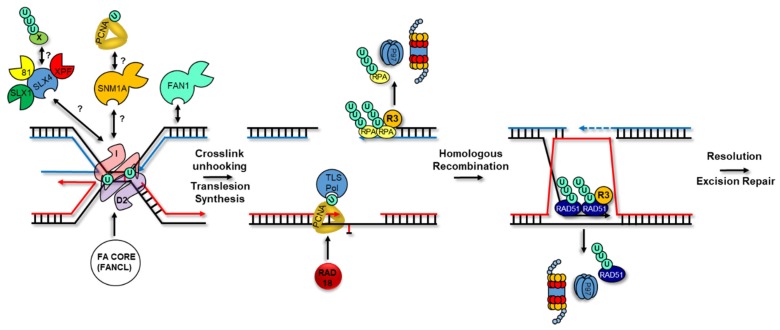
Ubiquitylation and the Fanconi Anemia Cross-link Repair Pathway. Upon cross-link detection, the FANCD2/I (D2/I) complex is activated by ATR phosphorylation and FA core complex ubiquitylation. This ubiquitylation promotes the cross-link unhooking step via the recruitment of nucleases containing ubiquitin binding-domains. These nucleases include SLX1, XPF and MUS81 (81) which are bound to the scaffold SLX4 ubiquitin-binding protein, SNM1A and FAN1. The mode of recruitment of SLX4 to ICLs is currently unclear and may depend on still unknown ubiquitylated factors (X). After lesion unhooking is completed, translesion polymerases are recruited to synthesize DNA on the parental duplex containing the lesion and the double-stranded break on the other duplex is resected to promote HR. RFWD3 (R3) associates with and ubiquitylates the ssDNA-binding RPA and the RAD51 recombinase and promotes their VCP/p97 and proteasome-dependent removal and degradation to drive HR to completion.

Activation of the FA pathway is triggered by ATR-mediated phosphorylation and subsequent mono-ubiquitylation of the FANCD2/I dimer by the core FA ubiquitin ligase complex following ICL detection [211,212,213]. The FA core complex contains the FANCL RING E3 ubiquitin ligase which works with its partner E2 UBE2T to mono-ubiquitylate FANCD2 on lysine 561 and FANCI on lysine 523 [214,215,216,217]. Activation of the FANCD2/I complex at ICLs is also regulated by the FAAP20 accessory subunit of the FA core complex that contains a UBZ domain with preference for K63-linked ubiquitin chains. FAAP20 is recruited via its UBZ domain to ICLs and promotes chromatin loading of the core complex in response to cross-linking agents [218,219,220]. The recognition of RNF8-UBC13 axis-mediated ubiquitylation products on chromatin by the UBZ domain of FAAP20 was proposed to be required for full activation of the FA pathway but the functional importance of the UBZ domain for ICL repair was inconclusive in one of the studies [218,220]. Once activated, mono-ubiquitylated FANCD2/I then guides the recruitment of structure-specific endonucleases to incise in 5′ and 3′ of the covalently linked nucleotides which results in cross-link unhooking (Figure 4). Among the enzymes that have been implicated in ICL repair, three are associated with the SLX4/FANCP nuclease scaffold/cofactor protein: XPF/ERCC4/FANCQ-ERCC1, MUS81-EME1 and SLX1 [221,222,223,224,225,226,227,228,229]. Cumulative evidence suggests that XPF-ERCC1 is the prevailing player in this process with smaller contributions from the other nucleases [230,231,232,233]. SLX4 orchestrates the activity of endonucleases by recruiting them to different types of lesions [234]. In the case of ICLs, SLX4 is tethered via direct binding of mono-ubiquitylated FANCD2/I and/or another ubiquitylated protein via its ubiquitin-reading dual UBZ domains (UBZ1 appears to have a predominant role in this) which enables the unhooking of the cross-link by XPF-ERCC1 [230,232,235,236,237,238]. Functionally, mutation/deletion of the UBZ domains of SLX4 causes FA, hypersensitivity to ICL agents and abrogation of its recruitment to psoralen-UVA micro-irradiation tracks in human cells and MMC-induced foci in DT40 chicken cells [221,227,235,236]. The presence of dual UBZ domains on SLX4 along with an *in vitro* preference of these domains for K63-linked ubiquitin chains suggest that poly-ubiquitylated proteins could also help mediate its recruitment to cross-linked DNA [227,236].

In addition to the SLX4 nuclease organizer, other ubiquitin-reading nucleases function in ICL repair. For instance, FAN1, a 5′ flap endonuclease and 5′-3′ exonuclease tethered to mono-ubiquitylated FANCD2/I via a UBZ domain was shown to be required for optimal resistance to cross-linking agents [239,240,241,242]. Despite strong molecular evidence supporting its implication in ICL repair, mutation of FAN1 in humans does not lead to FA but causes karyomegalic interstitial nephritis (KIN) and increased susceptibility to colorectal cancer [243,244,245,246,247]. Kidney defects are also observed in FAN1 KO or nuclease-dead knock-in mouse models [243,244,248]. Cells from KIN patients with mutations in FAN1 are sensitive to MMC but when treated with diepoxybutane do not show chromosomal breaks or cell cycle arrest as is typical for FA-patient cells [246]. Additionally, in DT40 cells, FAN1 deletion shows additive impairments in ICL repair with the rest of the FA pathway and this is also observed in co-depletion experiments in human cells suggesting that FAN1 participates in repair of ICLs in a unique manner [239,246]. Even more surprisingly, the mitomycin C sensitivity of KIN patient cells or FAN1 KO cells can be rescued by a UBZ-defective mutant but not by a nuclease-dead mutant indicating that FAN1’s role in ICL repair depends on its catalytic activity but not on its interaction with mono-ubiquitylated FANCD2 [244,246,249]. A UBZ FAN1 mutant is also still able to rapidly localize to psoralen-UV-A micro-irradiation sites via its SAP DNA-binding domain to mediate its ICL repair function [244]. More recently, it was shown that chromosomal abnormalities caused by HU-induced replication stress in MEFs cannot be rescued by a FAN1 UBZ mutant implicating this nuclease in other aspects of the RSR. Double nuclease-defective FAN1 and FANCD2 KO MEFs did not display additive levels of radial structures and chromatid breaks supporting the idea that FAN1 and mono-ubiquitylated FANCD2 work together to restrain fork elongation during HU-induced replication stress [249,250]. Contrastingly, FAN1 plays a UBZ domain- and FA core complex-independent role in replication fork restart in response to APH and in the absence of FANCD2 its unchecked activity can lead to degradation of APH-stalled forks [251]. Interestingly, G-quadruplex stabilization or UV-treatment was shown to induce UBZ- and PIP-box-mediated FAN1 interaction with ubiquitylated PCNA indicating that different RSR platforms can share ubiquitin-reading effectors to promote genomic integrity in a context-specific manner [252]. Altogether, these data support ubiquitin-dependent and -independent roles for the FAN1 nuclease in the RSR.

Apart from FAN1, *in vitro* nuclease assays have shown that the 5′-3′ exonuclease SNM1A/DCLRE1A (Sensitive to Nitrogen Mustard 1A/DNA Cross-link Repair 1A) can also perform unhooking of ICL lesions [253,254,255]. In humans, SNM1A is part of a family of three similar nucleases from the β-CASP metallo-β-lactamase group with SNM1B/Apollo and SNM1C/Artemis [256]. SNM1A KO mice or SNM1A-depleted human cells exhibit increased sensitivity to MMC and SNM1A or SNM1B disruption lead to cross-linking agent sensitivity in a non-epistatic way in DT40 cells [255,257,258]. SNM1C KO in DT40 cells does not induce ICL sensitivity [257]. Moreover, SNM1A was the only member of the family able to complement the ICL agent sensitivity of the *pso2* budding yeast mutant [259]. In fission yeast, SpPso2 (SNM1A homolog) and SpFan1 (FAN1 homolog) function in redundant pathways that support ICL repair [260]. This functional redundancy is conserved in mammals as well [244]. SNM1A can also collaborate with XPF-ERCC1 to resolve ICLs before they generate DSBs induced by the MUS81-EME1-mediated processing of stalled forks [255,261]. *In vitro*, processing of ICLs in replication fork-like structures bearing nascent leading strands by XPF-ERCC1 and SNM1A is stimulated by RPA [262]. Like FAN1, SNM1A possesses UBZ and PIP box domains that are both required for its recruitment to lesions by ubiquitylated PCNA in response to MMC or UV [263]. However, the functional significance of UBZ-dependent recruitment of SNM1A for cross-link repair has not yet been examined.

Following cross-link unhooking by nucleases, the lesion bypass step of the FA pathway requires the recruitment of translesion polymerases to replicate across the cross-link remnant. In vertebrates, REV1, REV3 and REV7 are proposed to be the main players during ICL repair [122,264,265,266,267,268]. TLS is orchestrated by mono-ubiquitylated PCNA and a ubiquitin-binding motif in REV1 may bring it to ICLs [269]. The current model posits that REV1 recruits POL ζ at the unhooked cross-link to promote the extension step of the TLS reaction [268]. Very recently, biallelic inactivating mutations in *REV7/MAD2L2/FANCV* were found to cause FA [270]. Interestingly, one of the mutations falls within the HORMA domain of REV7, known to mediate its interaction with REV1 and REV3L suggesting that the incapacity of patient cells to form an active Pol ζ is responsible for the defect in ICL repair [271]. The TLS-independent function of REV7 as part of the end-joining SHIELDIN complex that controls the extent of DNA resection at DSBs could suggest defects in downstream ICL repair steps in *REV7* mutant cells as well [272,273,274,275,276,277,278,279]. Finally, the recent implication of the RFWD3 ubiquitin ligase in the FA pathway supports roles for ubiquitylation beyond cross-link unhooking, nuclease recruitment and translesion synthesis into the HR-dependent steps of ICL repair [68,93,94].

## 5. De-Ubiquitylation and Replication Stress

De-ubiquitylation of the RSR platforms and more generally of genome maintenance factors is also a critical regulatory aspect of DNA damage signaling and repair (reviewed in [280,281]). Removal of ubiquitin moieties from their targets is carried out by multiple cysteine proteases known as de-ubiquitinating enzymes (DUBs). Here, we present the key DUBs that function on PCNA, FANCD2/I and RPA.

The USP1/UAF1 complex is an important de-ubiquitylase for the RSR that can remove ubiquitin from PCNA and the FANCD2/I complex. It was first identified via a targeted siRNA screen as the DUB that works on FANCD2 and subsequently found to target FANCI as well [216,282]. Disruption of USP1 in DT40 cells or in mice leads to cross-linker sensitivity and epistasis for ICL repair was shown between chicken *USP1* and *FANCI*. In mice, double FANCD2/USP1 KO leads to additive mitomycin C sensitivity indicating at least partly independent ICL repair functions for these 2 proteins [283,284]. In USP1^−/−^ MEFs, spontaneous and damage-induced FANCD2 ubiquitylation were increased. Moreover, FANCD2 foci assembly and HR were also defective. Similar ICL sensitivity and HR defects were also observed in UAF1^−/+^ MEFs [285]. The recruitment mechanism of USP1/UAF1 onto FANCD2/I involves the UAF1 subunit of the complex which contains SLDs at its C-terminus. These SLDs are bound by a SIM on FANCI which is critical for FANCD2/I de-ubiquitylation [286]. It was also recently shown by elegant *in vitro* experiments that FANCD2/I de-ubiquitylation occurs when DNA is disengaged which would correlate with the completion of DNA repair *in vivo* [287].

USP1 also targets PCNA during replication stress. Exposure of cells to UV irradiation induces auto-cleavage of USP1 which is subsequently degraded by the proteasome. This decrease in USP1 activity allows PCNA ubiquitylation levels to increase and promote TLS [288]. Interestingly, similar to the tethering of USP1/UAF1 onto FANCD2/I, the UAF1 SLDs mediate its recruitment onto PCNA via a SIM on ELG1, suggesting that USP1/UAF1 may coordinate HR and TLS [286]. Apart from USP1, ubiquitin-specific protease 10 (USP10) is another negative regulator of ubiquitylated PCNA. In this case, USP10 specifically recognizes PCNA that is decorated by ubiquitin but also by ISG15 (Interferon-stimulated gene 15, a ubiquitin-like molecule). USP10 activity on PCNA promotes the release of Pol η and terminates TLS [289]. In both situations, PCNA de-ubiquitylation signals the completion of DNA repair and allows the resumption of DNA replication.

More recently, a novel class of DUB was identified and shown to play a role in the RSR. Three groups simultaneously identified ZUFSP (zinc finger with UFM1-specific peptidase domain protein) as a DUB with a high preference for long K63-linked ubiquitin chains [290,291,292]. ZUFSP interacts with and cleaves K63-linked chains via tandem ubiquitin-binding domains and a c-terminal C78 papain-like peptidase domain. It co-localizes with RPA-ssDNA at micro-irradiation stripes and at FokI-induced DSBs and its recruitment is mediated by ubiquitin-binding domains and a series of zinc fingers at its N-terminus. KD of UBC13 strongly decreases ZUFSP accumulation at damage sites further supporting the role of K63-linked ubiquitin chains in this process [290]. An interaction with RPA was also reported by all three studies but RPA depletion did not impair the recruitment of ZUFSP to damage sites [96,290,291]. In agreement with a role for ZUFSP in the RSR, its KD increased the duration of S-phase and enhanced micronuclei formation in HU-treated cells. Prevention of micronuclei formation required the catalytic activity, the ubiquitin-binding domains and the N-terminal zinc fingers of ZUFSP [290]. Increased sensitivities to CPT and γ-irradiation along with spontaneous formation of γ-H2A.X and 53BP1 foci were also observed upon ZUFSP depletion further supporting its genome maintenance roles [291]. Interestingly, ZUFSP inhibition increased the levels of HU-induced and basal K63-linked ubiquitylation on RPA70, RPA32 suggesting that it may curb ubiquitylation on the RPA-ssDNA platform [96]. Additional work will be required to identify the full substrate complement of this enzyme during replication stress and to better understand its role(s) in the RSR.

## 6. Conclusions and Future Perspectives

Over the last few decades, the combined efforts of many research groups working with a variety of different model organisms have shown that the ubiquitin system coordinates the selection and guidance of DNA repair activities on RPA-ssDNA, PCNA and FANCD2/I, in a highly context-specific manner to safeguard the genome. At the same time, extensive crosstalk was documented between these platforms which by no means evolve in silos. Case in point, RPA-ssDNA generated rapidly at stalled forks promotes PCNA and FANCD2/I ubiquitylation by recruiting Rad18-Rad6 and by activating the ATR-ATRIP kinase respectively [28,115]. At the same time, PCNA can promote RPA-ssDNA formation by enhancing EXO1-mediated resection and many more connections exist between the three RSR comrades in arms [293]. RSR platforms also share ubiquitin-binding effectors as is the case for the nuclease FAN1 that is tethered to both ubiquitylated PCNA or FANCD2/I. The influence that these platforms have on each other and how DNA repair is achieved in a mostly error-free manner despite this extensive interplay will be important topics of future research.

In addition to the numerous E3 ubiquitin ligases discussed here, others for which substrates are still poorly characterized have strong experimental support for roles in the RSR and more ligases that function in DNA damage repair are still being discovered [294,295,296,297,298]. Delineating how these novel factors fit in the larger picture of the RSR will require the development of high-throughput substrate identification methods to define their ubiquitylomes and help us understand the full extent of their genome maintenance functions. With so many ligases converging to adjacent or even to the same RSR platforms, an outstanding question is how do different ligases that act on similar sets of substrates coordinate their activities to promote genome stability? A complete and fulfilling answer to this question will require *in vitro* reconstitution and careful examination of these ubiquitylation reactions.

Finally, impairment of the RPA, PCNA and FANCD2/I platforms by pharmacological means has the potential to synergize with current RSR-targeting drugs such as gemcitabine, ATR, CHK1 and WEE1 inhibitors [24,299]. Already, inhibitors of RPA DNA-binding and protein-protein interactions have shown promise *in vitro* and in cell models [300,301,302]. Peptides that impede the interaction of PCNA-binding proteins with their target also show cancer-specific cytotoxicity [303,304]. Furthermore, targeting the FA pathway by siRNA or small molecules increases cancer cell response to cisplatin and gemcitabine [305,306]. Understanding the complex exchange of factors that occurs at the surface of these genome maintenance platforms and its regulation by ubiquitin writers, readers and erasers, will help us develop complementary strategies to impair RSR activation on its main hubs and hopefully sensitize cancer cells even more to chemo- and radio-therapies.

## Abbreviations

4-NQO	4-Nitroquinoline
AAD	ATR-activating domain
APH	Aphidicolin
BRCT	BRCA1 C Terminus
CPD	Cyclobutane thymine-thymine dimer
CPT	Camptothecin
DCLRE1A	DNA Cross-link Repair 1A
DSB	Double-stranded DNA break
DUB	Deubiquitylating enzyme
ETAA1	Ewing’s Tumor Associated Antigen 1
FA	Fanconi anemia
HIRAN	HIP116 and RAD5 N-terminus
HLTF	Helicase-like transcription factor
HR	Homologous recombination
HU	Hydroxyurea
ICL	Inter-strand cross-link
IR	Irradiation
ISG15	Interferon-stimulated gene 15
KD	Knockdown
KIN	Karyomegalic interstitial nephritis
KO	Knockout
MEF	Mouse embryonic fibroblast
NZF	NPL4 zinf finger
OB	Oligonucleotide/oligosaccharide binding
PARI	PCNA-associated recombination inhibitor
PCNA	Proliferating Cell Nuclear Antigen
PIP	PCNA interacting-peptide
PTM	Post-translational modification
RING	Really Interesting New Gene
RPA	Replication Protein A
RSR	Replication stress response
SHPRH	SNF2 histone-linker PHD-finger RING-finger helicase
SIM	SUMO-interacting motif
SLD	SUMO-like domain
SNM1A	Sensitive to Nitrogen Mustard 1A
STUbL	SUMO-targeted ubiquitin ligase
SUMO	Small ubiquitin-like modifier
TLS	Translesion synthesis
TOPBP1	Topoisomerase II Binding protein 1
TS	Template switch
UBM	Ubiquitin-binding motif
UBZ	Ubiquitin-binding zinc finger
USP1	Ubiquitin specific protease 1
USP10	Ubiquitin specific protease 10
VCP	Vasolin-containing protein
WRNIP1	WRN-interacting protein 1
ZRANB3	Zinc finger RAN-binding domain containing 3
ZUFSP	Zinc finger with UFM1-specific peptidase domain protein
